# A Call to Action to Increase Uptake of Follow-Up Colonoscopy After Initial Positive Stool-Based Colorectal Cancer Screening

**DOI:** 10.1089/pop.2023.0199

**Published:** 2023-11-24

**Authors:** A. Mark Fendrick, John B. Kisiel, Durado Brooks, Vahab Vahdat, Chris Estes, Derek W. Ebner, Paul Limburg

**Affiliations:** ^1^ Division of General Medicine, Department of Internal Medicine, University of Michigan, Ann Arbor, Michigan, USA.; ^2^ Division of Gastroenterology and Hepatology, Department of Medicine, Mayo Clinic, Rochester, Minnesota, USA.; ^3^ Exact Sciences Corporation, Madison, Wisconsin, USA.

Guidelines for average-risk colorectal cancer (CRC) screening endorse several noninvasive modalities, including stool-based options. The 2021 US Preventive Services Task Force CRC recommendations state that positive results on stool-based screening tests require follow-up with colonoscopy,^1^ as patients with a positive test who do not undergo a follow-up colonoscopy have a 1.8-fold increased risk of CRC over a 6-year period.^2^ Nevertheless, a recent retrospective analysis demonstrated room for improvement in follow-up colonoscopy rates, especially among Black or Asian populations.^3^ The follow-up colonoscopy rate within 1 year after a positive stool-based test was only 56% in the total study population and differed by test type (48.7% after positive fecal immunochemical test [FIT]; 66.6% after positive multitarget stool DNA [mt-sDNA]).^3^

Financial barriers are a likely contributor to low follow-up colonoscopy completion rates. A 2021 study reported that 48% of commercially insured and 78% of Medicare beneficiaries paid nontrivial out-of-pockets costs for follow-up colonoscopy.^4^ Thus, in an effort to promote higher CRC screening completion rates and reduce disparities, federal regulations require commercial insurers and Medicare to eliminate out-of-pocket costs for follow-up colonoscopy as of January 2023. The objective of this analysis was to estimate the clinical and financial outcomes of this policy change.

Using the validated Colorectal Cancer and Adenoma Incidence and Mortality Microsimulation Model,^5^ a cohort of average-risk individuals who received mt-sDNA or FIT at age 45 for commercially insured patients and at age 65 for Medicare beneficiaries was modeled. For only those simulated patients with a positive mt-sDNA or FIT test, 2 scenarios were analyzed: (1) received follow-up colonoscopy ($0 cost sharing) or (2) no follow-up colonoscopy. The payer costs of follow-up colonoscopy were assumed to be $2,057 ($2,816 with polypectomy) for commercially insured patients and $1,118 ($1,624 with polypectomy) for Medicare beneficiaries.^6^ The clinical inputs for colonoscopy, mt-sDNA, and FIT sensitivity, specificity, and complication risk, as well as cost and utility inputs are listed in Supplementary Table S1. Outcomes measured included life-years gained (LYG), number of colonoscopies, CRC incidence and mortality reduction, quality-adjusted life years, and total lifetime costs. Total lifetime costs include screening costs, costs for complications from colonoscopy, and CRC medical costs.

Commercially insured and Medicare patients undergoing follow-up colonoscopy after a positive stool-based screening test experienced more LYG per 1000 individuals (83 and 80, respectively, with mt-sDNA; 43 and 46, respectively, with FIT; Table 1) compared with those who did not undergo follow-up colonoscopy. The commercially insured and Medicare patients undergoing a follow-up colonoscopy also experienced reduced CRC incidence (14% and 19% reduction, respectively, with mt-sDNA; 7% and 9% reduction, respectively, with FIT) and CRC-related mortality (16% and 23% reduction, respectively, with mt-sDNA; 8% and 12% reduction, respectively, with FIT; Table 1).

**Table 1.** Differences in Estimated Outcomes and Costs of Individuals with Positive MT-sDNA Test or FIT Who Undergo a Follow-Up Colonoscopy versus No Follow-Up Colonoscopy

Positive test: population	Δ LYG*^a^*	Δ CRC incidence reduction*^a^* (%)	Δ CRC mortality reduction*^a^* (%)	Δ COL^b^	Δ Screening cost^b,c^	Δ Colonoscopy complication cost^b^	Δ CRC-Medical cost^b^	Δ Total cost^b,d^
mt-sDNA: Commercially insured	83	14	16	2.47	$4,177	$105	−$8,891	−$4,609
mt-sDNA: Medicare	80	19	23	2.28	$2,669	$236	−$7,659	−$4,755
FIT: Commercially insured	43	7	8	2.72	$4,545	$126	−$11,110	−$6,439
FIT: Medicare	46	9	12	2.35	$2,768	$239	−$7,762	−$4,755

^a^ Per 1000 individuals.

^b^ Lifetime per person.

^c^ Screening costs include cost of stool-based CRC test, follow-up colonoscopy, repeat colonoscopy if full reach not achieved, surveillance, and symptomatic colonoscopy.

^d^ Total costs include screening costs, costs for complications from colonoscopy, and CRC medical costs.

COL, number of colonoscopies; CRC, colorectal cancer; FIT, fecal immunochemical test; LYG, life-years gained; mt-sDNA, multitarget stool DNA.

The total lifetime cost per patient for those receiving follow-up colonoscopy compared with stool-test-positive patients who did not receive follow-up colonoscopy was $4,609–$6,439 less for commercially insured patients and $4,755 less for Medicare beneficiaries. Total spending for patients receiving follow-up colonoscopy after a positive stool-based test was cost-saving when compared with no follow-up colonoscopy as long as the colonoscopy costs were less than $6,448 for commercial plans and $5,652 paid by Medicare.

These modeling analyses demonstrate that follow-up colonscopy after a positive stool-based test is an essential and necessary step in realizing the clinical and economic benefits of CRC screening. The findings that those who undergo follow-up colonoscopy are estimated to have improved CRC-related outcomes and lower total medical expenditures compared with those who do not receive follow-up colonoscopy should compel stakeholders to further invest in patient education, navigation, transportation, and other support services that facilitate completion of the overall CRC screening process, along with ensuring adequate and efficient access to follow-up colonoscopy. Such investments are urgently needed and will undoubtedly yield positive impacts at multiple levels.

## Supplementary Material

Supplemental data
