# Supplementary material for: A Call to Action to Increase Uptake of Follow-Up Colonoscopy After Initial Positive Stool-Based Colorectal Cancer Screening
Source: Popul Health Manag. 2023 Nov 24;26(6):448–50. doi: 10.1089/pop.2023.0199 (PMC10698770; doi:10.1089/pop.2023.0199)
Supplement: Supplemental data [file Suppl_TableS1.docx]

## Supplemental Material

**Supplemental Table 1.** Model inputs for colonoscopy, mt-sDNA, and FIT test performance characteristics.

| Model input | **Value for Commercial Insurance** | | **Value for Medicare** | | **Source Reference** |
| --- | --- | --- | --- | --- | --- |
| Sensitivity for CRC |  | |  | |  |
| Colonoscopy^*^ | 0.95 | | 0.95 | | Knudsen et al^1^ |
| mt-sDNA^†^ | 0.94 | | 0.94 | |  |
| FIT^†^ | 0.74 | | 0.74 | |  |
| Sensitivity for adenomas 1-5 mm |  | |  | |  |
| Colonoscopy^*^ | 0.75 | | 0.75 | | Knudsen et al^1^ |
| mt-sDNA^†^ | 0.09^‡^ | | 0.09^‡^ | |  |
| FIT^†^ | 0.03^‡^ | | 0.03^‡^ | |  |
| Sensitivity for adenomas 6-9 mm |  | |  | |  |
| Colonoscopy^*^ | 0.85 | | 0.85 | | Knudsen et al^1^ |
| mt-sDNA^†^ | 0.33^‡^ | | 0.33^‡^ | |  |
| FIT^†^ | 0.14^‡^ | | 0.14^‡^ | |  |
| Sensitivity for adenomas ≥10 mm |  | |  | |  |
| Colonoscopy^*^ | 0.95 | | 0.95 | | Knudsen et al^1^ |
| mt-sDNA^†^ | 0.42 | | 0.42 | |  |
| FIT^†^ | 0.22 | | 0.22 | |  |
| Specificity |  | |  | |  |
| Colonoscopy^*^ | 0.86^§^ | | 0.86^§^ | | Knudsen et al^1^ |
| mt-sDNA^†^ | 0.91 | | 0.91 | |  |
| FIT^†^ | 0.97 | | 0.97 | |  |
| Reach, % |  | |  | |  |
| Colonoscopy^*^ | 95 to end of cecum, remainder between rectum and cecum | | 95 to end of cecum, remainder between rectum and cecum | | Knudsen et al^1^ |
| mt-sDNA^†^ | Whole colorectum | | Whole colorectum | |  |
| FIT^†^ | Whole colorectum | | Whole colorectum | |  |
| Risk of complications (serious GI, other GI, and CV complications) |  | |  | |  |
| Colonoscopy^*^ | Age-specific risks | | Age-specific risks | | Knudsen et al^1^ |
| Screening test costs, $ |  | |  | |  |
| mt-sDNA | $508.87 | | $508.87 | | Protecting Access to Medicare Act pricing |
| FIT | $18.05 | | $18.05 | |  |
| Colonoscopy without polypectomy | $2,057 | | $1,118 | | Fisher et al 2022^2^ ‖ |
| Colonoscopy with polypectomy | $2,816 | | $1,624 | |  |
| Colonoscopy complications costs, $ |  | |  | |  |
| Gastrointestinal | $12,817.12 | | $9,287.76 | | Hathway et al, 2020^3 ¶^ |
| Serious gastrointestinal | $36,539.47 | | $26,477.88 | |  |
| Cardiovascular | $16,432.33 | | $11,907.49 | |  |
| CRC medical care costs, $ |  | |  | |  |
| Stage I |  | |  | | Mariotto et al, 2020^4^ ^¶^ |
| Initial care | $66,720.18 | | $48,347.95 | |  |
| Continuous care | $6,847.79 | | $4,962.16 | |  |
| Terminal care, CRC death | $134,642.03 | | $97,566.69 | |  |
| Terminal care, non-CRC death | $35,352.73 | | $25,617.92 | |  |
| Stage II |  | |  | |  |
| Initial care | $94,620.69 | | $68,565.72 | |  |
| Continuous care | $7,984.90 | | $5,786.16 | |  |
| Terminal care, CRC death | $151,781.23 | | $109,986.40 | |  |
| Terminal care, non-CRC death | $38,043.06 | | $27,567.43 | |  |
| Stage III |  | |  | |  |
| Initial care | $137,456.12 | | $99,605.88 | |  |
| Continuous care | $12,370.14 | | $8,963.87 | |  |
| Terminal care, CRC death | $158,908.81 | | $115,151.31 | |  |
| Terminal care, non-CRC death | $52,022.01 | | $37,697.11 | |  |
| Stage IV |  | |  | |  |
| Initial care | $204,265.97 | | $148,018.82 | |  |
| Continuous care | $58,563.10 | | $42,437.03 | |  |
| Terminal care, CRC death | $199,816.17 | | $144,794.32 | |  |
| Terminal care, non-CRC death | $125,139.79 | | $90,681.01 | |  |
| Health state utility | EQ-5D population norms | | EQ-5D population norms | | Szende 2014^5^ |
| Utility loss per event |  | |  | |  |
| Colonoscopy | −0.0055 | | −0.0055 | | Goede et al, 2017^6^ |
| Complication from colonoscopy, any | −0.0384 | | −0.0384 | |  |
| Utility loss per person per year of CRC care | Stage I-III | Stage IV | Stage I-III | Stage IV |  |
| Initial care | −0.15 | −0.34 | −0.15 | −0.34 | Goede et al, 2017^6^ |
| Continuous care | −0.10 | −0.29 | −0.10 | −0.29 |  |
| Terminal care, CRC death | −0.29 | −0.29 | −0.29 | −0.29 |  |
| Terminal care, non-CRC death | −0.10 | −0.29 | −0.10 | −0.29 |  |

CRC, colorectal cancer; CV, cardiovascular; EQ-5D, EuroQol 5 Dimension; FIT, fecal immunochemical test; GI, gastrointestinal; mt-sDNA, multi-target stool DNA test.

^*^Within reach, per lesion. The same test characteristics were assumed for screening, diagnostic follow-up, and surveillance colonoscopies.

^†^Per person.

^‡^For persons with 1 to < 6 mm adenomas, CRC-AIM assumes that the sensitivity is equal to the positivity rate in persons without adenomas. The sensitivity for persons with 6 to <10 mm adenomas was chosen such that the weighted average sensitivity for persons with 1 to < 6 mm and with 6 to <10 mm adenoma(s) is equal to the sensitivity for non-advanced adenomas.

^§^The lack of specificity with colonoscopy reflects the detection of non-adenomatous polyps, which in the case of colonoscopy, leads to unnecessary polypectomy, which is associated with an increased risk of complications.

‖Commercial costs inflated to December 2022 dollars.

^¶^Costs are converted to commercial insurance costs using a mean ratio of 1.38 for commercial to Medicare payment rates. Inflated to August 2022 dollars.

## References

1. Knudsen AB, Rutter CM, Peterse EFP, et al. Colorectal cancer screening: An updated decision analysis for the U.S. Preventive Services Task Force. Rockville, MD; 2021. Available from: <https://www.uspreventiveservicestaskforce.org/uspstf/document/final-modeling-report/colorectal-cancer-screening> [Last Accessed; July 12, 2023].

2. Fisher DA, Princic N, Miller-Wilson L-A, et al. Healthcare costs of colorectal cancer screening and events following colonoscopy among commercially insured average-risk adults in the United States. Curr Med Res Opin 2022;38:427-434

3. Hathway JM, Miller-Wilson LA, Jensen IS, et al. Projecting total costs and health consequences of increasing mt-sDNA utilization for colorectal cancer screening from the payer and integrated delivery network perspectives. J Med Econ 2020;23:581-592

4. Mariotto AB, Warren JL, Zeruto C, et al. Cancer-Attributable Medical Costs for Colorectal Cancer Patients by Phases of Care: What Is the Effect of a Prior Cancer History? J Natl Cancer Inst Monogr 2020;2020:22-30

5. Szende A, Janssen B, Cabases J. Self-Reported Population Health: An International Perspective based on EQ-5D. Dordrecht (NL); 2014.

6. Goede SL, Rabeneck L, van Ballegooijen M, et al. Harms, benefits and costs of fecal immunochemical testing versus guaiac fecal occult blood testing for colorectal cancer screening. PLoS One 2017;12:e0172864
